# Educational level, prevalence of hysterectomy, and age at amenorrhoea: a cross-sectional analysis of 9536 women from six population-based cohort studies in Germany

**DOI:** 10.1186/1472-6874-14-10

**Published:** 2014-01-16

**Authors:** Andreas Stang, Alexander Kluttig, Susanne Moebus, Henry Völzke, Klaus Berger, Karin Halina Greiser, Doris Stöckl, Karl-Heinz Jöckel, Christa Meisinger

**Affiliations:** 1 Institut für Klinische Epidemiologie, Medizinische Fakultät, Martin-Luther-Universität Halle-Wittenberg, Magdeburger Str. 8, 06112 Halle (Saale) Halle, Germany; 2 Institut für Medizinische Epidemiologie, Biometrie und Informatik, Medizinische Fakultät, Martin-Luther-Universität Halle-Wittenberg, Halle, Germany; 3 Institut für Medizinische Informatik, Biometrie und Epidemiologie, Universitätsklinikum der Universität Duisburg-Essen, Essen, Germany; 4 Institut für Community Medicine, Ernst-Moritz-Arndt-Universität, Greifswald, Germany; 5 Institut für Epidemiologie und Sozialmedizin, Universität Münster, Münster, Germany; 6 Deutsches Krebsforschungszentrum (DKFZ), Abteilung Epidemiologie von Krebserkrankungen, Heidelberg, Germany; 7 Institute of Epidemiology II, Helmholtz-Zentrum München, German Research Center for Environmental Health (GmbH), Neuherberg, Germany

**Keywords:** Hysterectomy, Population surveillance, Prevalence, Education, Amenorrhoea

## Abstract

**Background:**

Hysterectomy prevalence has been shown to vary by education level. Hysterectomy influences age at amenorrhoea. The aim of this study was to examine these associations in Germany within population-based data sets.

**Methods:**

Baseline assessments in six population-based cohorts took place from 1997 through 2006 and included 9,548 women aged 20–84 years. All studies assessed hysterectomy history, school and professional degrees. Degrees were categorized into three levels each. Adjusted prevalence ratios and 95% confidence intervals (95% CI) were estimated.

**Results:**

Prevalences were higher in West Germany than East Germany, increased by age, and leveled off starting at 55–64 years. The age- and study-adjusted prevalence ratio (lowest versus highest school level) was 2.61 (95% CI: 1.28-5.30), 1.48 (95% CI: 1.21-1.81), and 1.01 (95% CI: 0.80-1.28) for women aged 20–45, 45–64, and 65 and more years respectively. The estimated adjusted prevalence ratios per one unit decrement of the educational qualification score (range 1 = lowest, 8 = highest) were 1.29 (95% CI: 1.02-1.64), 1.08 (95% CI: 1.04-1.12), and 0.98 (95% CI: 0.93-1.03) for women aged 20–44, 45–64, and 65–84 years respectively. Age at amenorrhoea was on average 6.2 years lower (43.5 years versus 49.7 years) among women with a history of hysterectomy than those without.

**Conclusions:**

Lower educational level was associated with a higher hysterectomy prevalence among women aged 20–64 years. Several mediators associated with educational level and hysterectomy including women’s disease risk, women’s treatment preference, and women’s access to uterus-preserving treatment may explain this association. At population level, hysterectomy decreases the age of amenorrhoea on average by 6.2 years.

## Background

Hysterectomy is the most frequently performed major surgical procedure in gynaecology in many countries of the world [1]. Several nationwide studies on indications for hysterectomy revealed that at least 80% of hysterectomies are performed for the treatment of benign diseases of the female genital tract [2,3]. Comparisons of population-based hysterectomy rates for benign diseases of the genital tract across regions including Germany have repeatedly shown considerable regional variation [4,5].

Large regional variation within countries cannot be explained by gynaecological need alone, giving rise to concerns about unnecessary surgery that may expose women to unnecessary risks. In this context, there are concerns that women of lower educational level have a higher risk of hysterectomy than women of higher educational level. Hysterectomy has been shown to vary along a number of social factors including school education, occupational status, household income, and adult and childhood socioeconomic position [6]. Hysterectomy prevalences are typically higher among women with lower educational level in several countries. The association between hysterectomy prevalence and educational level may depend on birth cohort with stronger inverse associations among younger cohorts [6].

In 1990, the former German Democratic Republic (GDR) and the Federal Republic of Germany (FRG) were reunified. Whereas the GDR with its centralistic health care system did not provide financial incentives for physicians to perform hysterectomy, the FRG provided financial incentives. Reunified Germany has a comprehensive health care insurance system: people below a certain income threshold (about 90%) are health insured by the sickness fund system by mandatory law in Germany; people above this threshold (about 10%) can choose to be insured by the sickness fund system or purchase a private insurance. However, educational level may still be associated with the hysterectomy prevalence. For example, the clinician’s willingness to offer uterus-preserving therapy to women with benign diseases of the genital tract may depend on educational level of the women; furthermore, the attitude of women with benign diseases of the genital tract regarding uterus-preserving therapies may differ by educational level. Age at amenorrhoea is substantially influenced by a history of hysterectomy. The British Women’s Heart and Health Study revealed that self-reported age at menopause was 51.5 years and 45.9 years among women without and with a history of hysterectomy and/or bilateral ovariectomy [7].

The aim of this study was to examine the association between educational level and prevalence of hysterectomy in the general population in West and East Germany by use of available population-based cross-sectional data from six prospective population-based cohort studies including more than 9536 women. Furthermore, we wanted to study the influence of hysterectomy on age at amenorrhoea at population level.

## Methods

We searched for available German population-based cohort studies that enabled the study of the cross-sectional association between educational level and prevalence of hysterectomy. Eligible studies had to be finished by October 2011 and study data had to be available by January 2012. We searched for relevant studies in publications of the annual meetings of German epidemiological and social medicine societies. With the exception of the Gutenberg Health Study, all identified population-based cohort studies performed in Germany fulfilled the inclusion criteria.

We collected data from six German population-based cohort studies that were conducted between 1997 and 2006. All studies draw random samples from mandatory lists of residence that are considered the most complete lists of residents in Germany. Baseline response across these studies ranged between 55.8% and 76%. All studies either included a questionnaire or interview related to hysterectomy or gynaecologic surgery: the KORA-S4 study and KORA-F3 study in Bavaria, South Germany [8], the Heinz Nixdorf Recall Study (HNR) [9] and the Dortmund Health Study (DHS) [10], both in the Ruhr area of North Rhine-Westphalia (West Germany), the Study of Health in Pomerania (SHIP-0) (East Germany), [11] and the Cardiovascular Disease, Living and Ageing in Halle Study (CARLA) in Saxony-Anhalt (East Germany) [12].

KORA-S4 was approved by “Ethikkommission der Bayerischen Landesärztekammer”, AZ 99186, January 25, 2001. KORA-F3 was approved by “Ethikkommission der Bayerischen Landesärztekammer”, AZ 03097, June 21, 2004. HNR was approved by “Ethikkommission der Medizinischen Fakultät Essen”, AZ 99-69-1200, May 12th, 1999, DHS was approved by “Ethikkommission der Ärztekammer Westfalen-Lippe und der Medizinischen Fakultät der Universität Münster”, AZ 3VIIBerger, September 22, 2003, CARLA was approved by “Ethikkommission der Medizinischen Fakultät der Universität Halle-Wittenberg”, AZ Prof.Sz-B, SHIP-0 was approved by “Ethikkommission des Landes Mecklenburg-Vorpommern an der Universität Greifswald”, AZ BB 39/08, May 15, 2008. All participants gave written informed consent. Core study characteristics are summarised in Table 1.

**Table 1:** Study characteristics across six population-based cohort studies in Germany 1997-2006

	**West Germany**				**East Germany**	
	**KORA-S4 study**	**KORA-F3 study**	**HNR study**	**DHS study**	**CARLA study**	**SHIP-0 study**
Study region	Augsburg	Augsburg	Essen, Mülheim, Bochum	Dortmund	Halle (Saale)	West Pomerania
Inhabitants per km^2^ of the regions	107	107	2,571	2,107	1,756	52
Federal State of Germany	Bavaria	Bavaria	North Rhine-Westphalia	North Rhine-Westphalia	Saxony-Anhalt	Mecklenburg-West Vorpomerania
Population-based hysterectomy rate (per 100,000)^1^	347	347	369	369	382	436
Recruitment period	1999-2001	2004-2005	2000-2003	2003-2004	2002-2006	1997-2001
Baseline response (%)	66.8	76	55.8	66.9	64.1	68.8
Participating women (N)	2,171	1,639	2,038	694	810	2,184
Age range (years)	25-74	35-84	45-75	25-74	45-83	20-81
Highest school degree (%)^2^						
Low	53.3	59.1	64.1	50.4	32.0	37.4
Middle	27.6	26.6	20.0	22.2	51.9	47.8
High	18.9	13.9	15.7	26.4	16.1	14.4
Missing	0.2	0.4	0.2	1.0	0.1	0.4
Highest professional degree (%)^2^						
Low	19.6	21.3	18.7	18.6	10.6	8.8
Middle	69.3	69.5	70.6	63.0	76.3	75.8
High	11.2	8.8	10.5	15.3	7.2	12.9
Missing^3^	0.2	0.4	0.3	3.2	5.9	2.5
Educational qualification (two-dimensional score)						
1 (=lowest)	0.0	0.0	1.6	2.3	1.7	0.9
2	15.4	16.2	14.7	12.7	5.3	7.2
3	36.2	43.5	47.4	37.3	23.5	26.5
4	21.6	19.2	17.8	19.9	39.5	32.1
5	7.6	6.6	1.9	0.9	8.3	14.8
6	7.8	5.2	5.9	10.7	8.6	3.4
7	11.2	8.8	2.8	0.4	2.0	4.3
8 (=highest) ^4^	0.0	0.0	7.7	15.3	5.3	8.6
Missing	0.2	0.4	0.3	0.6	5.8	2.2
Median (P10; P90)	3 (2; 7)	3 (2; 6)	3 (2; 7)	3 (2; 7.5)	4 (3; 6)	4 (3; 7)

1) Age-standardised hysterectomy rates per 100,000 women per year in the Federal States for any indication according to the national hospitalisation file; age standard: female population in Germany at December 31, 2005; for details see [5]; 2) for details, see method section; 3) Higher proportion of missing data due to women who currently undergoing professional re-orientation; 4) DHS study did not distinguish between professional degrees from universities and applied universities; these women (n = 106) were assigned a score of 8; P10: 10^th^ percentile; P90: 90^th^ percentile.

With the exception of two cohort studies, all studies assessed hysterectomies by a few questions: “Has your uterus been removed? If yes, at which age?”, (CARLA, DHS, KORA-F3, KORA-S4). However, SHIP-0 and HNR used the following wordings: SHIP: “Have you ever undergone gynaecologic surgery? If yes, which type of surgery was it?”, HNR: “Has there been any disease-related reason, why your menstruation stopped? If yes, what was the reason?”. Reported gynaecologic surgeries in these two studies were classified by trained clinicians (hysterectomy: yes/no). Women were asked whether they are menstruating. If they reported no, they were asked the age when the last menstrual period occurred (SHIP-0, CARLA, HNR). DHS, KORA-F3, KORA-S4 did not ask questions related to menstruation.

Educational level is a multifactorial construct that includes several dimensions like school education and professional degree after leaving school. School degrees can be re-translated into number of years of school education. All studies assessed these two factors. We categorised school education into three levels: low (no school leaving certificate or lower secondary school leaving certificate [*Hauptschulabschluss/Volksschulabschluss*], < 10 years school education), middle (intermediate secondary school leaving certificate [*Mittlere Reife/Polytechnische Oberschule*], 10 years school education), and high (upper secondary school leaving certificate [*Fachhochschulreife/Abitur*], 12–13 years school education). Of note, in Germany there are no formal degrees after 11 years of school. We classified the highest professional degree after leaving school on the basis of the German vocational training and education system: low (no professional degree), middle (in-company vocational training [*beruflich betriebliche Ausbildung*]), full-time vocational training [*beruflich schulische Ausbildung*], and high (including higher education, degree from engineering schools, and (applied) university degree [*(Fach) Hochschulstudium*]). In addition, we combined the school education and professional degree according to the recommendations of the German Society for Epidemiology to form a two-dimensional score called educational qualification score. The lowest score is 1 (no school degree and no professional degree) and the highest score is 8 (13 years school education and university degree) [13].

We excluded women from each cohort data set (usually below 3% of all women) with missing data on hysterectomy. In the HNR study, 15.3% of women had missing data on hysterectomy because during the baseline recruitment of that study, a shortened assessment program was run when study personnel was scant. For the comparison of prevalences across cohort studies, we estimated age-specific prevalences and exact 95% confidence intervals (95% CI). To estimate the confounder-adjusted association between educational level (school degree, professional education, two-dimensional educational qualification score) and prevalences of hysterectomy, we ran log-binomial regression models to estimate prevalence ratios and corresponding 95% confidence intervals and used disjoint indicator variables for the educational level variables [14]. In case of non-convergence of the models, we used Poisson regression with robust variance. We used the highest school degree, professional degree, and educational qualification score respectively as the reference groups for the analyses. We identified minimally sufficient adjustment sets using a diagram that represents the relations among the exposure, outcome, and other variables (available on request) [15]. Adjustment variables included age (continuous variable) and study (disjoint indicators). All analyses were done with SAS, version 9.3.

## Results

Age-specific prevalences of hysterectomy were lower in former East than West Germany especially among women aged 45 and more years (prevalence ratios East versus West: 20–44 years: 1.18, 95% CI 0.72-1.94; 45–64 years: 0.69, 95% CI 0.61-0.79; 65–84 year: 0.62, 95% CI 0.52-0.72). Prevalences of hysterectomy increased by age: all studies showed some levelling off of the prevalence starting at age 55–64 years (Additional file 1: Figure S1).

The association between education and hysterectomy prevalence depended on age. Among women aged 20–45 years and 45–64 years, hysterectomy prevalences tended to be higher among women with lower school degrees, lower professional degrees (Table 2), and with a lower two-dimensional educational qualification score (data not shown). Among women aged 65 years and more, there was barely any association between school degree, professional degree, educational qualification score and hysterectomy prevalence. East German studies (CARLA and SHIP-0) tended to show weaker associations between education and hysterectomy prevalence than West German studies (Table 2).

**Table 2:** Hysterectomy prevalences and highest school and professional degree

	**KORA-S4**		**KORA-F3**		**HNR**		**DHS**		**CARLA**		**SHIP-0**	
Women (N)	2,171		1,639		2,038		694		810		2,184	
Hysterectomised N (%)	406	(18.7)	388	(23.7)	496	(24.3)	151	(21.8)	174	(21.5)	235	(10.8)
**School degree % and 95% CI**												
< 45 year												
Low	4.7	2.6-7.8	5.6	2.3-11.2			7.5	2.1-18.2			1.8	0.0-9.7
Middle	2.5	1.1-4.8	3.3	0.9-8.2			0.0	0.0-4.3			2.7	1.6-4.2
High	1.1	0.2-3.1	0.0	0.0-3.9			2.5	0.5-7.1			1.6	0.3-4.7
45-64 years												
Low	31.6	27.8-35.5	28.7	24.5-33.1	25.8	22.9-28.8	31.0	24.6-37.9	24.1	13.5-37.6	20.6	16.8-24.9
Middle	27.9	21.9-34.5	21.9	16.8-27.8	21.4	17.0-26.4	32.8	21.6-45.7	19.2	14.9-24.0	13.1	9.6-17.3
High	17.3	10.6-26.0	14.4	8.6-22.1	14.9	10.8-19.7	14.8	6.6-27.1	14.0	7.7-22.7	18.2	11.5-26.7
65+ years												
Low	32.7	27.2-38.6	35.0	30.3-39.9	29.5	25.2-34.0	41.0	31.3-51.3	23.9	18.2-30.3	15.0	11.5-19.2
Middle	31.8	20.9-44.4	28.0	18.7-39.1	22.3	14.4-32.1	45.5	24.4-67.8	29.5	21.2-38.8	20.4	10.6-33.5
High	29.6	13.8-50.2	48.6	31.4-66.0	27.5	15.9-41.7	37.5	8.5-75.5	18.9	8.0-35.2	10.0	1.2-31.7
**Professional degree % and 95% CI**												
< 45 years												
Low	7.1	2.0-17.3	0.0	0.0-22.1			6.7	0.8-22.1			0.0	0.0-20.6
Middle	2.8	1.7-4.3	4.1	2.1-7.2			2.7	0.7-6.7			2.5	1.5-3.9
High	1.3	0.2-4.5	0.0	0.0-7.4			1.6	0.0-8.5			3.0	0.8-7.4
45-64 years												
Low	32.1	25.7-39.2	31.1	23.1-40.2	29.3	22.8-36.5	27.8	16.5-41.6	12.5	2.7-32.4	21.2	12.1-33.0
Middle	30.0	26.4-33.8	24.2	20.8-27.9	23.2	20.7-25.8	30.7	24.5-37.3	20.4	16.4-24.9	17.3	14.5-20.5
High	13.2	6.5-22.9	18.0	10.6-27.5	14.7	10.0-20.5	10.5	2.9-24.8	15.0	5.7-29.8	15.2	9.4-22.7
65+ years												
Low	33.5	26.5-41.1	27.0	21.2-33.4	30.0	23.7-36.9	35.6	21.9-51.2	19.4	10.4-31.4	10.6	5.6-17.8
Middle	31.6	25.0-38.7	40.6	34.8-46.5	26.9	22.4-31.8	46.1	34.5-57.9	25.2	19.9-31.1	17.7	13.5-22.5
High	27.3	6.0-61.0	37.5	15.2-64.6	31.8	13.9-54.9	20.0	0.5-71.6	16.7	3.6-41.4	13.6	2.9-34.9

Percentages express the prevalence of hysterectomy; 95% CI: exact 95 percent confidence interval.

**Figure 1:**
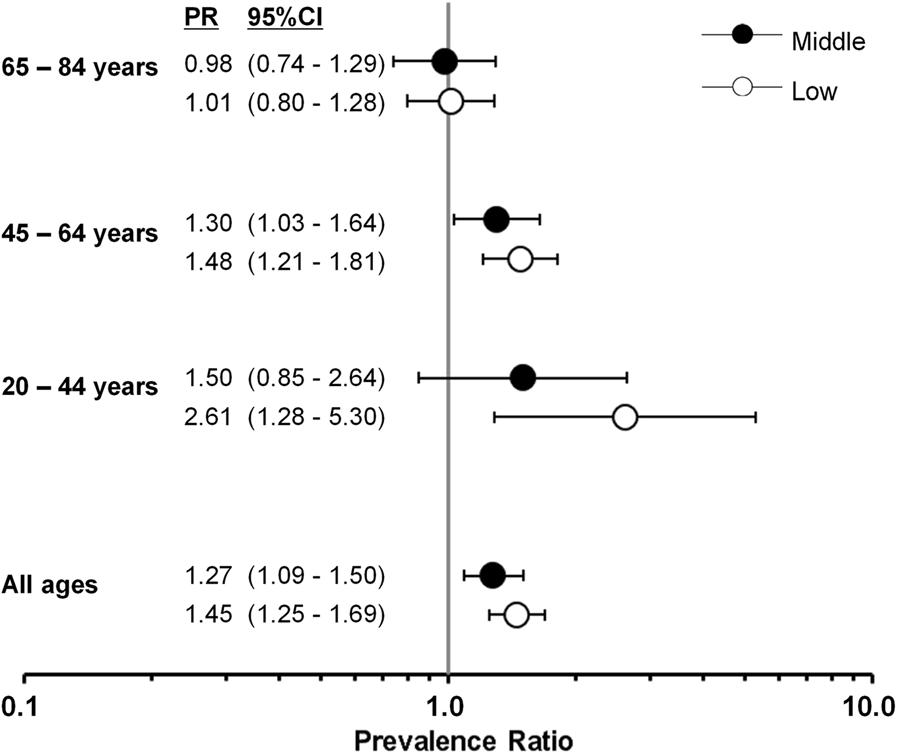
**Estimated adjusted prevalence ratios of hysterectomy and school degree among 9536 women of six German cohort studies, 1997–2006.** All estimated prevalence ratios (PR) are adjusted for study and age (metric variable); school education: low (no school leaving certificate or lower secondary school leaving certificate), middle (intermediate secondary school leaving certificate), and high (upper secondary school leaving certificate).

**Figure 2:**
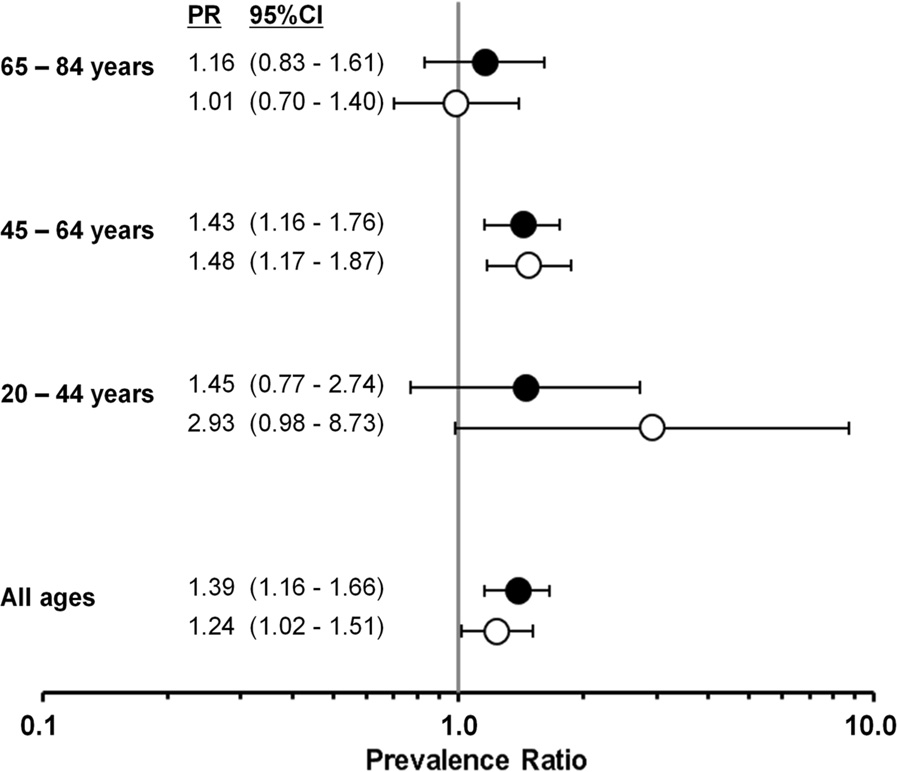
**Estimated adjusted prevalence ratios of hysterectomy and professional degree among 9536 women of six German cohort studies, 1997–2006.** All estimated prevalence ratios (PR) are adjusted for study and age (metric variable); professional education: low (including no professional degree), middle (in-company vocational training, full-time vocational training), and high (including higher education).

The age- and study-adjusted prevalence ratios were highest among women with the lowest school degree followed by middle school degree within the age range of 20–64 years. For example, the adjusted prevalence ratio was highest among women aged 20–44 years with the lowest school education (prevalence ratio 2.61, 95% CI: 1.28-5.30). Among women aged 65 years and more, there was virtually no association between school education and hysterectomy prevalence (Figure 1). We observed a similar pattern for the association between professional education and hysterectomy prevalence (Figure 2).

**Figure 3:**
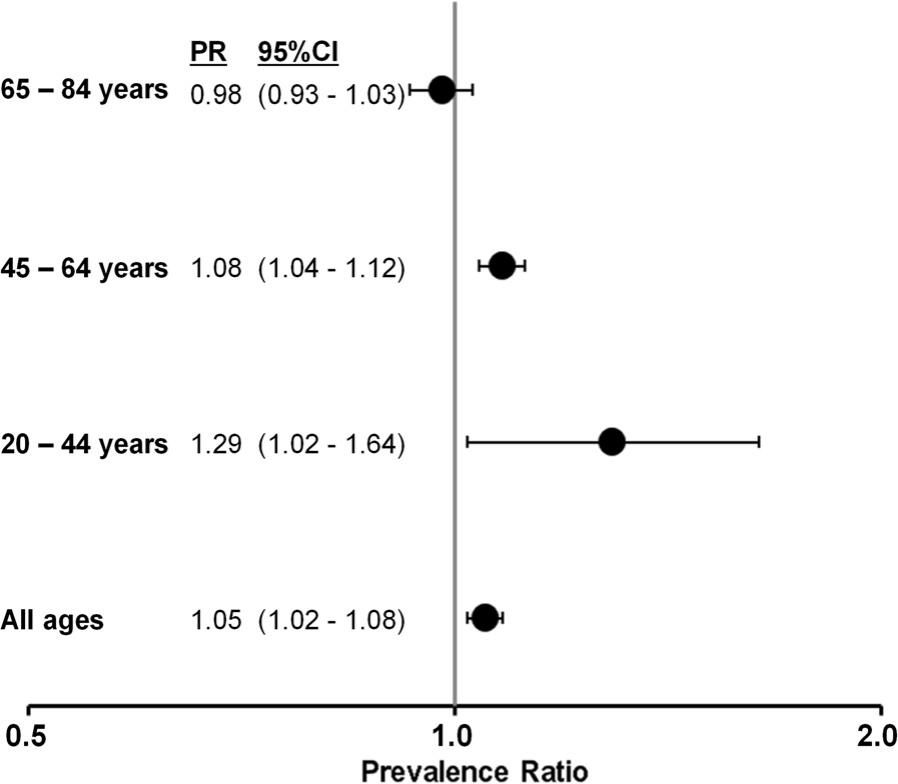
**Estimated adjusted prevalence ratios of hysterectomy and two-dimensional educational qualification score among 9536 women of six German cohort studies, 1997–2006.** All estimated prevalence ratios (PR) are adjusted for study and age (metric variable); prevalence ratios expressed per one unit decrement of the two-dimensional educational qualification score that ranged between 1 = lowest and 8 = highest.

**Figure 4:**
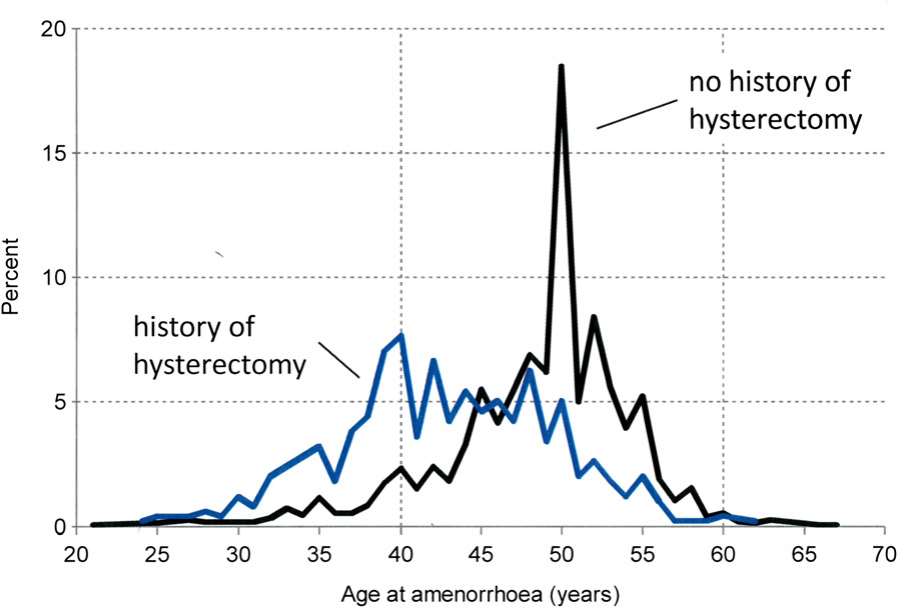
**Age (years) at amenorrhoea by history of hysterectomy among German cohort studies, 1997–2006.** Relative frequency of women with amenorrhoea by 1-year age groups; mean age and standard deviation among women without a history of HE 49.7 years (5.2) and with a history of HE 43.5 years (7.0) with an estimated mean difference of 6.2 years (95% CI: 5.8-6.6). Contributing studies to this pooled analysis were HNR, CARLA, and SHIP-0.

The estimated adjusted prevalence ratios per one unit decrement of the educational qualification score (range: 1–8) were 1.29 (95% CI: 1.02-1.64), 1.08 (95% CI: 1.4-1.12), and 0.98 (95% CI: 0.93-1.03) for women aged 20–44, 45–64, and 65–84 years respectively (Figure 3). In the pooled analysis of the three studies that assessed age at amenorrhoea, the mean age at amenorrhoea was 43.5 years (standard deviation (SD): 7.0 years) and 49.7 years (SD 5.2 years) among women with and without hysterectomy respectively (mean difference 6.2 years, 95% CI: 5.8, 6.6 years) (Figure 4).

## Discussion

Hysterectomy prevalences were higher in West than East Germany. The association between education and hysterectomy prevalence depended on age. Among women aged 20–45 years and 45–64 years, hysterectomy prevalences were lower among women with the highest school and higher professional education. Among women aged 65 years and more, there was barely any association between school degree, professional education, two-dimensional educational qualification score, and hysterectomy prevalence.

Similarly as in our study, international studies found an inverse association between educational level variables including school and professional degree [16,17], employment status, [17] and hysterectomy prevalence or cumulative risk. Women with benign diseases of the female genital have typically a choice between a variety of uterus-preserving therapies and hysterectomy. For example, in cases of genital prolapse without uterine pathology, one should first explore options for conservative treatment or uterus-sparing surgery (fixation of uterus or vagina) before considering hysterectomy (S1 guideline on genital prolapse) [18]. Women with uterine adenomyosis who still want children or prefer to preserve the uterus should initially be treated by means of interventional radiology procedures such as embolization and MRI-guided focused ultrasonographic ablation as part of a study, if at all possible. Alternatives to hysterectomy also include gestagens, hormonal contraceptives, and levonorgestrel intrauterine systems (S2 guideline on diagnosis and treatment of endometriosis) [19]. In women with heavy menstrual bleeding, hysterectomy should be considered only when other treatment options have failed, are contraindicated, or are refused by the patient, or when the patient expressly requests hysterectomy (NICE guideline) [20]. Myomectomy and uterine artery embolization are viewed as safe and effective alternatives to hysterectomy in patients with symptomatic leiomyoma (guideline of the American College of Obstetricians and Gynecologists) [21].

Educational level may influence, that is mediate, the risk of hysterectomy through a number of pathways. For example, women of higher educational level usually have smaller numbers of pregnancies and a later completion of the desired family size than women of lower educational level. Women’s preferences (e.g. desire to preserve fertility) and depth of information given by clinicians [22] may play a role and form the basis of shared decision making (patient-doctor relation) [23]. A woman’s perception of the effect of hysterectomy and her access to less invasive surgical techniques, are other potential mediators. For example, Materia et al. speculated that highly educated women in Rome may have had better access to uterus-preserving therapies for removing leiomyoma compared to women with lower education who are more likely to undergo unnecessary hysterectomy irrespective of their reproductive age [24]. Alternatively, women of lower educational level may have a higher risk of benign diseases and illnesses of the genital tract that ultimately result in hysterectomy. Unfortunately, there are no incidence data available that show that women of lower educational level have a higher incidence of benign diseases of the genital tract.

Our observed age-specific dependence of the hysterectomy prevalence on school and professional degree is in line with a recent study from Australia and the UK: Cooper et al. found a stronger inverse association between educational level and hysterectomy rates in younger than older cohorts in both countries [6]. The lack of an association between educational level and hysterectomy prevalence in older women in Germany may reflect changes in attitudes especially regarding the treatment of benign diseases of the female genital tract in the 1980s. A recent nationwide analysis showed that the peak of the hysterectomy rate is at ages 44–47 in Germany [25]. Women aged 65 and more years who participated in the population-based cohort studies from 1997 through 2006 went through this critical age period in the late 1970s and 1980s when hysterectomy was most likely less frequently used for the treatment of benign diseases of the female genital tract as in later years. Unfortunately, population-based hysterectomy rates in Germany of the years 1970 through 2004 are not available. Furthermore, the associations between educational level and factors that increase the probability of hysterectomy like parity [26] and obesity [27] among women in their 40s may have been weaker in the late 1970s and 1980s than in the 2000s.

The observed prevalence differences between former East and West Germany may reflect differences in health care systems before reunification in 1990. As there were no financial incentives among physicians to perform hysterectomy in East Germany before 1990 (in contrast to West Germany), physicians of the German Democratic Republic may have been more reluctant to remove the uterus than physicians of the Federal Republic of Germany. This may explain why the association between school degree, professional degree and prevalence of hysterectomy was weaker in East than West German studies. Alternatively, educational level in former communist states generally played a less prominent role for shared decision making. Population-based studies revealed that the vast majority of hysterectomies are undertaken for the treatment of benign diseases of the female genital tract [2-4,28]. Therefore, our observed East–west difference of hysterectomy prevalence is mainly driven by different hysterectomy rates for benign indications that may either reflect East–west differences of the risk of benign diseases of the female genital tract or different opinions regarding the indication for hysterectomy. Unfortunately, there is no study available that provides evidence for this hypothesis. Finally, the introduction of the West German health care system to East Germany in 1990 may have changed attitudes towards hysterectomy in East Germany. A recent analysis of hysterectomy rates in 2005–2006 revealed that hysterectomy rates were higher in East than West Germany [5].

Age at amenorrhoea was on average 6.2 years lower among women with a history of hysterectomy similarly as in the British Women’s Heart and Health Study (5.6 years) [7]. Given the peak of hysterectomy rates at ages 44–47 for benign disease of the female genital tract in Germany [25], and an age at natural amenorrhoea (menopause) of 50 years, the observed age difference of 6.2 years is plausible.

There are some factors that limit our conclusions from the results. First, all prevalence estimates were based on self-report only. However, several reliability and validation studies revealed that self-reported hysterectomy has both a high reliability and validity [29]. A reliability study within the Nurses’ Health study revealed that age at menopause reported two times within two years agreed within one year for 95% of women reporting incident surgical menopause. For natural incident menopause, the reproducibility within one year was 82% [30]. Second, response proportions of the baseline recruitment of the cohort studies ranged between 55.8% and 76% and undersampling of women with a history of malignant diseases of the female genital tract is likely. For example, detailed analyses in the KORA-S4 study revealed that nonresponders included a higher percentage of people with impaired health and with greater behavioural health risks [31]. As a consequence, the prevalence of hysterectomy may be underestimated in our study. In addition, the strength of association between educational level and hysterectomy prevalence may have been underestimated as women who are too ill to participate also includes women with a history of cervical cancer, a cancer that typically has a higher incidence among women with lower educational level [32]. However, the proportion of hysterectomies for the treatment of malignant diseases of the female genital tract in the general population is usually below 20% which weakens this potential biasing effect [2,3]. Third, a survivor bias among elderly women could have produced the null result for the association between education and hysterectomy prevalence if hysterectomized women of lower education had a lower survival probability than women of higher education. Fourth, we could not distinguish between birth cohort and age effects related to the hysterectomy prevalence as the baseline assessments of the cohorts only span a narrow interval of calendar years (1997–2006).

## Conclusions

In conclusion, we found an inverse association between school degree, professional degree, two-dimensional educational qualification score and prevalence of hysterectomy among younger women in Germany. Several mediators associated with educational level and hysterectomy including women’s disease risk, women’s treatment preference, and women’s access to uterus-preserving treatment may explain this association. Prospective cohort studies that include the assessment of these mediating factors may provide further insights into the association between educational level and hysterectomy incidence. At population level, hysterectomy decreases the age of amenorrhoea on average by 6.2 years.

## Abbreviations

CARLA: Cardiovascular disease, living and ageing in Halle study; DHS: Dortmund health study; HNR: Heinz nixdorf recall study; KORA-S4: Kooperative gesundheitsforschung in der region Augsburg, S4; KORA-F3: Kooperative gesundheitsforschung in der region Augsburg, F3; PR: Prevalence ratio; SHIP-0: Study of health in pomerania; 95% CI: 95% confidence interval.

## Competing interests

The authors declare that they have no competing interests.

## Authors’ contributions

AS designed the pooling project and performed the statistical analyses. He drafted the manuscript. AK, SM, HV, KB, KHG, DS, KHJ and CM acquired and provided data of the cohort studies and helped to draft the manuscript. All authors read and approved the final manuscript.

## Pre-publication history

The pre-publication history for this paper can be accessed here:

http://www.biomedcentral.com/1472-6874/14/10/prepub

## Supplementary Material

Additional file 1: Figure S1 Age-specific (10-year groups) prevalences of hysterectomy among 9536 women of six German population-based cohort studies 1997–2006. 10-year age groups; whiskers show exact 95% confidence intervals. Click here for file
