# Supplementary figures and images for: Educational level, prevalence of hysterectomy, and age at amenorrhoea: a cross-sectional analysis of 9536 women from six population-based cohort studies in Germany
Source: BMC Womens Health. 2014 Jan 16;14:10. doi: 10.1186/1472-6874-14-10 (PMC3898063; doi:10.1186/1472-6874-14-10)

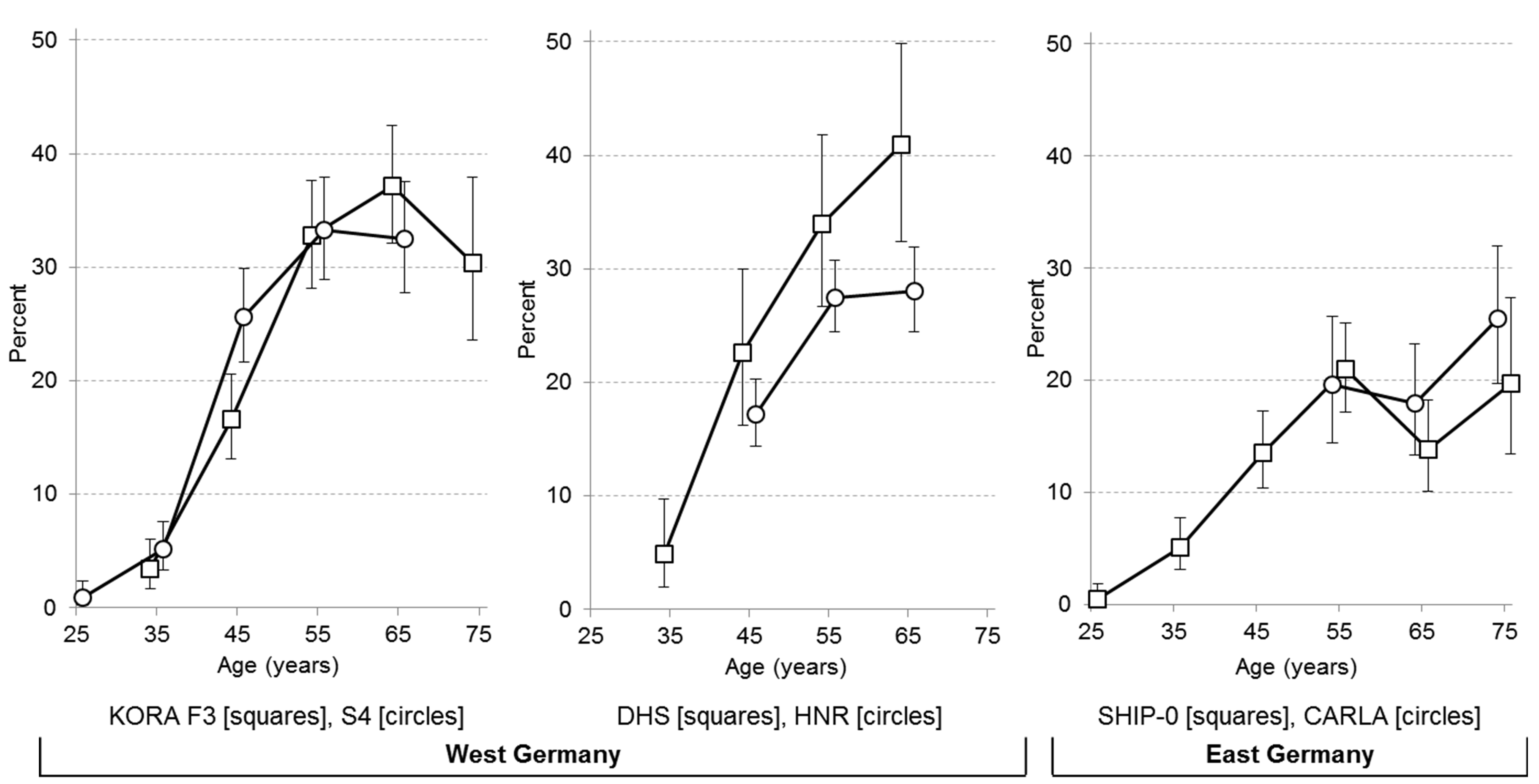

Supplement: Additional file 1: Figure S1 — Age-specific (10-year groups) prevalences of hysterectomy among 9536 women of six German population-based cohort studies 1997–2006. 10-year age groups; whiskers show exact 95% confidence intervals. [file 1472-6874-14-10-S1.tiff]
